# Effects of a web‐based relapse prevention program on abstinence: Secondary subgroup analysis of a pilot randomized controlled trial

**DOI:** 10.1002/npr2.12272

**Published:** 2022-06-11

**Authors:** Ayumi Takano, Yuki Miyamoto, Tomohiro Shinozaki, Toshihiko Matsumoto, Norito Kawakami

**Affiliations:** ^1^ Department of Mental Health and Psychiatric Nursing Tokyo Medical and Dental University Tokyo Japan; ^2^ Department of Psychiatric Nursing The University of Tokyo Tokyo Japan; ^3^ Department of Information and Computer Technology, Faculty of Engineering Tokyo University of Science Tokyo Japan; ^4^ Department of Drug Dependence Research National Center of Neurology and Psychiatry Tokyo Japan; ^5^ Department of Mental Health The University of Tokyo Tokyo Japan

**Keywords:** drug dependence, randomized controlled trial, relapse prevention, subgroup analysis, web‐based intervention

## Abstract

**Aim:**

The effect of a web‐based relapse prevention program might vary depending on a specific population if the study participants included drug users with various characteristics. This secondary analysis explored subgroups among Japanese drug users that may benefit from a web‐based relapse prevention program.

**Methods:**

Outpatients with drug use disorder (*n* = 48) were randomly assigned to an 8‐week, six‐session web‐based relapse prevention program (intervention group) or web‐based self‐monitoring only (control group). We tested the effects of the intervention on abstinence in different subgroups divided by a primary abused drug (methamphetamine vs other drugs), previous face‐to‐face relapse prevention (received vs not received), and outpatient treatment term (long‐term: ≥3 years vs short‐term: <3 years). Consecutive abstinence duration from the primary abused drug was compared in the subgroups, and the interaction between the intervention condition and the subgroup condition was assessed.

**Results:**

In the subgroup with short‐term outpatient treatment, the intervention group maintained better abstinence than the control group. For those who used methamphetamine or those who had previously received a face‐to‐face relapse prevention program, the intervention group showed larger effect sizes than the results from all the participants. However, the interaction between the intervention condition and the subgroup condition was not significant for any subgroup.

**Conclusions:**

This study suggests that patients with short‐term treatment may benefit from a web‐based relapse prevention program as an alternative treatment. We need to recruit and allocate patients considering their treatment term in a future definitive trial.

## 1 INTRODUCTION

Internet‐ or smartphone‐based therapeutic interventions for dealing with illicit drug use and addictive behaviors have been developed and adapted to address treatment implementation challenges,^1^, ^2^ such as accessibility, confidentiality and stigmatization, and human‐resource limitations for treatment providers.^3^, ^4^, ^5^ Such tools are often based on existing face‐to‐face psychosocial approaches, such as cognitive behavioral therapy or community reinforcement approach.^1^ Some interventions were designed to be drug‐specific, such as for opioids, cannabis, or cocaine,^1^, ^6^ with functionalities and modules addressing a target population. However, most interventions were developed and adapted to the particulars of the United States or European culture.^1^, ^7^ In Asian countries, including Japan, methamphetamine is common among drug users, and domestic treatment programs have focused on methamphetamine users.^8^ Policies in Asian countries addressing illicit drugs are typically zero‐tolerance, and treatment is usually abstinence‐oriented. Thus, to address these particulars, a new web‐based program was needed to fit the Asia context and local treatment goals.

To the best of our knowledge, there was only one randomized controlled trial (RCT) that assessed the efficacy of web‐based intervention for drug users in Asia.^9^ This web‐based intervention, named e‐SMARPP, was developed as an adjunct treatment based on an existing relapse prevention program called SMARPP (Serigaya Methamphetamine Relapse Prevention Program), which was widely implemented in Japan.^10^ The existing relapse prevention program utilized the Matrix Model developed in the United States to treat mainly stimulant users.^11^ The Matrix Model is a packaged program constructed with treatment elements based on cognitive behavioral therapy and contingency management using detailed treatment manuals. It has demonstrated effectiveness for drug and alcohol reduction. The target population in the study was mostly methamphetamine users that had already received outpatient treatment and maintained reasonable abstinence. To assess the effectiveness of e‐SMARPP in the RCT, the intervention group received the complete content of e‐SMARPP for 8 weeks, including six relapse prevention sessions, self‐monitoring, and provision of relevant information.^9^ In the relapse prevention sessions, personalized feedback comments utilizing motivational interviewing techniques from a trained web‐therapist were provided after patients submitted activity assignments. The control group only received the self‐monitoring content embedded in the e‐SMARPP website because the data regarding self‐monitoring during the intervention was used as the outcomes. The primary outcome was abstinence duration during the intervention. Although there was no significant difference between groups because of the small sample size, the effect size was medium (Cohen’s *d* = 0.42).^9^ Effectiveness might vary depending on a specific population because the study participants included drug users who had various characteristics in terms of the type of drug and treatment history. Thus, it is helpful to identify patients who benefit from a web‐based program when considering the trial design in a future definitive trial or when choosing a suitable treatment for each patient.

This secondary subgroup analysis of the RCT aimed to examine details regarding the effects of the web‐based relapse prevention program developed for Japanese drug users. We looked at the type of primary drug and treatment history through three subgroups because these variables were considered to influence outcomes such as abstinence or completion rate of the intervention: primary drug (methamphetamine or other drugs), previous face‐to‐face relapse prevention (received or not received), and outpatient treatment term (short‐term or long‐term).

## 2 METHODS

### 2.1 Design

This study was a secondary data analysis of a pilot RCT that assessed the effects of the web‐based relapse prevention program.^9^ The methods and main results of the original RCT are described in detail in our publications.^9^, ^12^ We conducted a two‐arm, parallel‐group, and rater‐blinded RCT. This study protocol was registered with the University Hospital Medical Information Network clinical trial registry (UMIN000016075). The Ethics Committee of the University of Tokyo and each trial site approved this study protocol.

### 2.2 Participants and definition of the subgroups

We recruited outpatients with a diagnosis of drug use disorders (abuse/dependence) assessed by DSM‐IV or DSM‐5 from six psychiatric hospitals in Japan. We included outpatients who had used any illicit drug or abused prescription drugs in the past year. Their treatment history was not considered. The participants accessed the e‐SMARPP website via PC, smartphone, or tablet devices.

In total, 48 patients entered the RCT (intervention group: *n* = 23, control group: *n* = 25). There were no significant differences in participant characteristics at the baseline between the intervention group and the control group. We included 44 patients with complete data regarding daily drug use or abstinence during the intervention to this secondary analysis (intervention group: *n* = 19, control group: *n* = 25). We divided patients by primary drug (methamphetamine or other drugs), previous face‐to‐face relapse prevention (received or not received), and outpatient treatment term (long‐term: ≥3 years or short‐term: <3 years). Other drugs included any substances other than alcohol and tobacco (eg, cocaine, MDMA, prescription drugs). The third subgroup was divided based on the median of the outpatient term.

### 2.3 Measures

The longest duration of consecutive abstinence and proportion of complete abstinence from the primary drug during the 8‐week intervention (56 days) were assessed as outcomes using self‐monitoring in a calendar format, similar to previous studies.^13^, ^14^ Demographic variables included age of first drug use, and severity of drug dependence at baseline. Drug dependence severity was assessed using the Japanese version of the Drug Abuse Screening Test (DAST‐20).^15^, ^16^ The total score ranges from 0 to 20, and a high score represents a severe condition. The cutoff scores for low (1–5), intermediate (6–10), substantial (11–15), and severe (more than 16) were based on recommendations in previous studies.^17^, ^18^, ^19^

### 2.4 Statistical analyses

The abstinence duration from the primary drug during the intervention was compared in each subgroup using *t* test. The effect size (Cohen’s *d*) of 0.2, 0.5, and 0.8 were considered small, medium, and large effects, respectively.^20^ The interaction between the intervention and the subgroup was assessed using a two‐way analysis of variance (ANOVA). The effect size of the interaction and the main effects in the ANOVA model were shown using eta squared. Since the purpose of the subgroup analyses was to determine intervention effects in each subgroup, we conducted a t test and calculated the effect sizes for each subgroup analysis regardless of the test results of the interaction. We also compared the proportion of complete abstinence during the intervention by subgroups using a chi‐squared test. SPSS Statistics ver. 25 was used for all statistical analyses.

## 3 RESULTS

In the subgroups, the number of participants was almost even between the intervention group and the control group, and participant characteristics were nearly the same between the groups (Tables S1, S2, and S3). Table 1 shows the results of the subgroup analysis assessing the efficacy of e‐SMARPP on abstinence duration from the primary drug during the intervention. Among participants with short‐term outpatient treatment, the intervention group showed a longer abstinent duration than the control group (*P* = 0.04, *d* = 0.96). The proportion of complete abstinence in the intervention group was significantly higher than in the control group (100.0% vs 58.3%, *P* = 0.02). For those who used methamphetamine or those who had previously received a face‐to‐face relapse prevention program, the intervention group showed larger effect sizes than those in all the participants. In these subgroups, the proportion of complete abstinence in the intervention group was higher than in the control group. However, the interactions between the intervention condition and the subgroup condition were not significant in all subgroups. Detailed ANOVA results are shown in Tables S4, S5, S6.

**TABLE 1:** Subgroup analyses on abstinence during the intervention (56 days)

	*n*	Abstinent duration from the primary drug					Interaction (intervention*subgroup)		Complete abstinence during the intervention		
		Mean	SD	*t*	*P*	*d*	*F*	*P*	*n*	%	*P*
All participants											
I	19	48.8	14.7	1.45	0.16	0.42			15	78.9	0.18
C	25	41.2	20.3						15	60.0	
Primary abused drug											
Methamphetamine											
I	13	51.5	11.2	1.66	0.12	0.71	0.85	0.36	11	84.6	0.11
C	11	40.0	20.7						6	54.5	
Other drugs											
I	6	42.8	20.4	0.76	0.94	0.03			4	66.7	0.92
C	14	42.1	20.7						9	64.3	
Previous face‐to‐face relapse prevention											
Received											
I	10	52.5	11.1	1.39	0.18	0.54	0.15	0.70	9	90.0	0.23
C	13	42.9	21.7						9	69.2	
Not receive											
I	9	44.7	17.7	0.65	0.53	0.29			6	66.7	0.45
C	12	39.3	19.4						6	50.0	
Outpatient treatment term											
Long‐term: ≥3 years											
I	9	40.8	18.7	0.78	0.94	0.03	1.43	0.24	5	55.6	0.78
C	13	40.1	21.9						8	61.5	
Short‐term: <3 years											
I	10	56.0	0.0	2.46	0.04	0.96			10	100.0	0.02
C	12	42.3	19.2						7	58.3	

Abbreviations: I: intervention group (e‐SMARPP group), C: control group (self‐monitoring group).

## 4 DISCUSSION

The web‐based relapse prevention program achieved abstinence in some subgroups. Thus, the web‐based relapse prevention program might benefit specific patients. Definitive conclusions were difficult regarding inter‐group differences because there was no significant interaction between the intervention condition and the subgroup and due to the small sample size; however, it is necessary to consider how the web‐based program might be most helpful in terms of specific patient characteristics when developing trial design in a future definitive RCT or choosing suitable treatment for patients with various backgrounds.

Outpatients in the intervention group with a short‐term treatment maintained longer abstinence than in the control group. In this population, most participants already maintained complete abstinence at the baseline and maintained abstinence during the intervention. Meanwhile, outpatients in the intervention group with long‐term treatment did not improve abstinence, and the effect size in this subgroup was smallest compared to those in other subgroups. This intervention might be useful for maintaining abstinence in outpatients who started treatment within the last couple of years, but not for those who had already received treatment for more than 3 years. Participants who had received long‐term treatment may have other factors requiring long‐term treatment, such as psychiatric comorbidity or persistent drug dependence. An additional integrated intensive treatment modality rather than a simple web‐based program may be needed for patients with complicated problems.^21^

Outpatients with methamphetamine problems could benefit from the web‐based relapse prevention program because methamphetamine users showed a larger effect size than all participants. Originally, relapse prevention programs were developed for stimulant users, and thus, were likely readily applicable to Japanese methamphetamine users. This study included patients who used new psychoactive substances and prescription drugs such as benzodiazepines. It is more challenging to treat these patients using web‐based programs because of the variety of drugs, severe intoxication or withdrawal symptoms, and limited evidence regarding treatment.^22^ Accordingly, in future studies, it is important to focus on specific drug users when developing and providing web‐based relapse prevention.

For outpatients who had already received face‐to‐face relapse prevention, although they showed a slightly larger effect size than all participants, it was unclear that an additional web‐based relapse prevention program had a booster effect. Some studies have assessed the effects of booster sessions of cognitive behavioral therapy and motivational interviewing on psychiatric symptoms such as depression or problem gambling, but the results were inconclusive.^23^, ^24^, ^25^, ^26^, ^27^ The results in our study might be influenced by a ceiling effect rather than a booster effect for patients with the previous relapse prevention program because most patients were already maintaining abstinence before the intervention. Thus, the effectiveness of an additional internet‐based treatment program for people with drug problems is still unclear.

There were several limitations to this study. First, the sample size for the subgroup analysis was small, and participants in the subgroups were not allocated to the intervention randomly. Second, it might be challenging to detect significant differences between the groups because of the active control condition. Third, the reliability of the collected data was uncertain because all variables were self‐reported.

## 5 CONCLUSIONS

This study suggests that patients with short‐term treatment benefit from a web‐based relapse prevention program as an alternative treatment. It is necessary to recruit and allocate patients considering their treatment term in a future definitive RCT that evaluates the effectiveness of the program. Further research with a large sample size is needed to determine subgroups that benefit more from the program.

## AUTHOR CONTRIBUTIONS

AT, NK, YM, and TS designed the original protocol for this study. AT and TM developed the intervention program, recruited patients, and collected data. AT and TS conducted the statistical analyses. AT and NK drafted the manuscript. All authors read and approved the final manuscript to be published.

## CONFLICT OF INTEREST

The authors declare no conflict of interest.

## APPROVAL OF THE RESEARCH PROTOCOL BY AN INSTITUTIONAL REVIEW BOARD

The Ethics Committee of the University of Tokyo and each trial site (National Center of Neurology and Psychiatry, Saitama Psychiatric Medical Center, Kanagawa Psychiatric.

Medical Center, Okayama Psychiatric Medical Center, Tokyo Metropolitan Matsuzawa Hospital, and APARI clinic) approved this study protocol.

## INFORMED CONSENT

Face‐to‐face informed consent was conducted using explanation documents, and signed consent forms were obtained from all participants.

## REGISTRY AND REGISTRATION NO. OF THE STUDY/TRIAL

University Hospital Medical Information Network clinical trial registry (UMIN000016075).

## ANIMAL STUDIES

None.

## Supporting information


Appendix S1

## Data Availability

The datasets in this study are not available, because we have not obtained the agreement of participants to disclose raw data nor the approval of the ethics committee in each institution for data sharing.
